# Brain–Immune Interactions and Neuroinflammation After Traumatic Brain Injury

**DOI:** 10.3389/fnins.2019.01178

**Published:** 2019-11-12

**Authors:** Virginie Dinet, Klaus G. Petry, Jerome Badaut

**Affiliations:** ^1^INSERM U1029, Angiogenesis and Neuroinflammation Group, University of Bordeaux, Bordeaux, France; ^2^CNRS UMR 5287, INCIA, Brain molecular Imaging Team, University of Bordeaux, Bordeaux, France; ^3^Department of Basic Sciences, Loma Linda University School of Medicine, Loma Linda, CA, United States

**Keywords:** traumatic brain injury, neuroinflammation, complement, blood-brain barrier, astrocyte

## Abstract

Traumatic brain injury (TBI) is the principal cause of death and disability in children and young adults. Clinical and preclinical research efforts have been carried out to understand the acute, life-threatening pathophysiological events happening after TBI. In the past few years, however, it was recognized that TBI causes significant morbidity weeks, months, or years after the initial injury, thereby contributing substantially to the overall burden of TBI and the decrease of life expectancy in these patients. Long-lasting sequels of TBI include cognitive decline/dementia, sensory-motor dysfunction, and psychiatric disorders, and most important for patients is the need for socio-economic rehabilitation affecting their quality of life. Cerebrovascular alterations have been described during the first week after TBI for direct consequence development of neuroinflammatory process in relation to brain edema. Within the brain–immune interactions, the complement system, which is a family of blood and cell surface proteins, participates in the pathophysiology process. In fact, the complement system is part of the primary defense and clearance component of innate and adaptive immune response. In this review, the complement activation after TBI will be described in relation to the activation of the microglia and astrocytes as well as the blood–brain barrier dysfunction during the first week after the injury. Considering the neuroinflammatory activity as a causal element of neurological handicaps, some major parallel lines of complement activity in multiple sclerosis and Alzheimer pathologies with regard to cognitive impairment will be discussed for chronic TBI. A better understanding of the role of complement activation could facilitate the development of new therapeutic approaches for TBI.

## Traumatic Brain Injury

Traumatic brain injury (TBI) is a consequence of a direct or indirect external mechanical impact on the brain inducing the disruption of the normal structure and function of the brain. Despite its simple mechanical definition, TBI is a very complex and heterogeneous injury with various severities graded in clinic. The severity of TBI is predicated on alteration of consciousness, organized according to the Glasgow Coma Scale (GCS) for assessment of neurological function and neuroimaging as mild, moderate, or severe (Maas et al., 2008). TBI is a major burden worldwide, and in the United States, it represents more than 30% of all injury-related deaths (Faul et al., 2010). In Europe, it is about 75,000 deaths each year (Maas et al., 2015), and it affects all ages. It is important to underline that pediatric and elderly populations are more vulnerable than adults (Faul et al., 2010). Therefore, TBI has an economic toll on the society that includes medical expenses and losses of productivity (Corso et al., 2006; Gustavsson et al., 2011). A widespread research on TBI pathophysiology is still sparse compared to other brain diseases such as Alzheimer's disease (AD) and the progressive chronicity of multiple sclerosis (MS).

Pathophysiology following TBI is grouped into the following: (1) The primary acute event is the mechanical injury that can give extracranial and intracranial complications like epidural hematoma, subarachnoid hemorrhage, or hypoxia. Thus, TBI is particularly heterogeneous depending on the primary injury severity, location, and the age and sex of the patient. Important in the TBI pathogenesis is that the functional outcomes may deteriorate over time after initial admission of the patient (Baethmann et al., 1988). For example, the patient can communicate early after TBI and then worsen with loss of consciousness, with an early small contusion that becomes a much larger injury the day after. This evolution is due to the secondary injury events (Plesnila, 2016). (2) The secondary injury cascade includes the following landmarks: glutamatergic excitotoxicity, calcium overload, vascular dysfunction, and inflammation/neuroinflammation. Inflammation/neuroinflammation can last for several months and could contribute to chronic TBI (Plesnila, 2016). The pattern of “chronic brain disease” has been leading the concept of accelerating of brain aging post-TBI (Johnson et al., 2010; Pop and Badaut, 2011; Smith et al., 2013). Patients can present long-lasting behavioral dysfunctions including some psychiatric disorders after TBI (Smith et al., 2013). Very recently, a nationwide population cohort study was done in Denmark to monitor individuals who had TBI from 1977 to 2013. Over the study period, 4.7% of recruited people had at least one TBI, and about 112,218 (85.0%) of 132,093 individuals had a mild TBI for their first TBI diagnosis; those people were 24% more likely to develop dementia than those without a history of TBI. Additionally, the younger the person was at the time of the injury, the higher the risk of dementia development over time (Fann et al., 2018).

Several animal models have been developed in order to extend our understanding of the cellular and molecular mechanisms in post-TBI secondary injuries (Prins and Hovda, 2003; Petraglia et al., 2014). Having a wide variety of preclinical models is a good sketch to mimic the heterogeneity in human TBI. However, it is important to highlight that the definition of the degree of TBI severity differs between animal models, and it is difficult to compare findings between models (Badaut et al., 2019). Sometimes, it is challenging to make the translation with clinical severities. The molecular and cellular mechanisms of secondary injuries are dependent on injury severity and brain location. The post-TBI outcome is more severe for younger patients; therefore, it is important to consider the age of the animal in the preclinical models (McMillan and Teasdale, 2007; Himanen et al., 2011; Pop and Badaut, 2011). In fact, neuroinflammation/inflammation response will vary in function of the location and severity of the impact as well as the age of the animal. The review focuses on some of the cerebrovascular alterations and the importance of the inflammation through the exploration of the complement system as a new potential mechanism. Actually, the role of the complement pathway in TBI pathogenesis is not yet well-understood.

## Neurovasculature Impairment Post-TBI

The neurovascular unit (NVU) is composed of neurons, a vascular tree with endothelial cells, smooth muscle cells, pericytes, astrocytes, and microglia (Zhang et al., 2012). Immune cells have been included as part of this physiological unit (Zhang et al., 2012). Cognitive impairments in AD as well as in healthy aging have been paralleled with NVU alterations (Iadecola, 2004; Raz et al., 2016). Similarly, NVU alteration is a typical landmark of the secondary injuries after TBI with, for example, a decrease of blood–brain perfusion with consequences on the good operation of the neurons (Pop and Badaut, 2011; Jullienne et al., 2016). In fact, preclinical TBI models unveil blood–brain barrier (BBB) disruption, hemorrhage, and cerebral blood flow alterations with hypo-perfusion, which are illustrations of the NVU alterations (Golding, 2002; DeWitt and Prough, 2003; Pop and Badaut, 2011). Cerebrovascular dysfunction has been described for almost two decades as a poor prognostic outcome. In fact, early decrease of the cerebral blood flow (CBF) post-injury induces hypoxic events in the brain tissue that directly contribute to the loss of neurons. In addition, the changes in BBB permeability play a role in edema formation with disruption of brain homeostasis. It exacerbates the cascade of secondary injury events including excitotoxicity (glutamate release and resulting higher metabolic demand) and inflammation (with the complement pathway, see below). TBI is a chronic brain disorder with molecular changes at the BBB several months after the initial injury (Jullienne et al., 2016). As it has been suggested in AD, the long-term alterations at the blood–brain interface could therefore be associated with premature aging of the brain after TBI (Pop and Badaut, 2011; Keightley et al., 2014; Jullienne et al., 2016).

### Brain Perfusion Alterations

Early decrease of CBF is a landmark of the pathophysiology post-TBI and its recovery represents a prognostic factor for outcome post-TBI (Bouma et al., 1991, 1992; DeWitt and Prough, 2003). Preclinical models appropriately reproduce CBF changes observed in clinic. Decreased CBF is observed after TBI using controlled cortical impact (CCI) and lateral fluid percussion (LFP) injury models and it is described up to 8 months after injury in adult rats (Bouma et al., 1991; Bryan et al., 1995; Plesnila et al., 2003; Hayward et al., 2010), which probably feeds the inflammatory process. Even for mild TBI, a decrease of CBF has also been described (Villapol et al., 2014; Long et al., 2015). In a mild CCI model, the reduction of CBF was described with restoration at 30 days post-TBI in young adult mice (Villapol et al., 2015). Importantly, hypo-perfusion adds to the development of the secondary injuries after TBI by decreasing glucose and oxygen delivery. Moreover, an ischemic event could be aggravated by the presence of cell hyper-metabolism during the first 6 h after concussion in rats (Yoshino et al., 1991). Consequently, decreased CBF and hyper-metabolism occurring early after TBI result in a mismatch between demand and supply, known as uncoupling of CBF and glucose metabolism, with direct consequence and aggravation of the secondary injuries and directly feeding the inflammation and neuroinflammation process. At the time, dysfunction in vascular perfusion is paralleled with BBB hyper-permeability and edema process.

### BBB Dysfunction Post-TBI

The presence of hemorrhage is a marker of acute vascular dysfunction, and it is frequently observed in moderate and severe TBI. BBB and cerebral blood vessel alterations are frequently shadowed by edema formation with dramatic consequences on morbidity and mortality because it induces intracranial hypertension and contributes to the vicious cycle of the secondary injury cascade (Unterberg et al., 2004). Even for mild TBI characterized by the absence of major bleeding, the BBB structure can be damaged or altered during the acute period after the injury. Very importantly, the BBB dysfunction is not only an acute event; it could also be observed several months and years after the traumatic event in human post-mortem tissue (Hay et al., 2015) and in preclinical models (Pop et al., 2013; Jullienne et al., 2014).

The consequences of primary injury on the BBB properties significantly differ according to the age, the type, and the severity of the lesion. The “opening” of the BBB can be observed as early as 3 min after injury in a model of concussion (Povlishock et al., 1978). The “opening” of BBB can be characterized with the presence of two types of lesions on the luminal surface of pial arterioles: crater-shaped indentations and dome-shaped projections of the endothelial cell surface, suggesting necrotic cells (Wei et al., 1980). The tight junction protein complex is potentially altered after TBI. In fact, pial, and intracerebral blood vessels demonstrate decreased claudin-5 and occludin expressions early after injury (Nag et al., 2007). Similar changes in tight junction protein complex have been described in rodent models of mild TBI induced by blast shock waves, with specifically decreased expression in occludin, claudin-5, and *zonula occludens* protein-1 at 6 h and 24 h post-TBI (Abdul-Muneer et al., 2013). The changes in tight junction proteins have been paralleled with an increase of immunoglobulin G extravasation from 5 min to 48 h post-injury (Yeoh et al., 2013; Shetty et al., 2014). However, it is important to underline that tight junction complexes appear intact under electron microscopy during the first hours after a mild TBI; then, the expression of the tight junction protein is altered (Rafols et al., 2007). Therefore, the changes in expression of the tight junction proteins and increased IgG extravasation are not directly linked with physical rupture of the tight junctions in electron microscopy (Knowland et al., 2014; Haley and Lawrence, 2017). Similarly, IgG extravasation is increased close to the site of impact and surrounding tissue in the absence of a direct relation with changes in claudin-5 expression in a model of juvenile CCI (Pop and Badaut, 2011; Badaut et al., 2015). Similarly, IgG extravasation has been observed in the corpus callosum at 24 h after mild closed head injury, suggesting BBB hyper-permeability even for mild injury (Rodriguez-Grande et al., 2018).

Changes of expression of the tight junction proteins are accompanied by up-regulation of various matrix metalloproteases (MMPs), which have been involved in BBB alteration. In fact, MMP-9 and MMP-2 increase acutely after TBI in rodents (Wang et al., 2000; Zhang et al., 2010). MMP-3 activity is increased chronically after TBI and possibly plays a role in synaptic remodeling (Zhang et al., 2010). Up-regulation of MMPs alters proteins of the extracellular matrix and participates in BBB alteration and neurovascular unit dysfunction.

In summary, it is important to highlight that neurovascular unit disturbance, encompassing BBB and blood flow changes, fuels the inflammation and neuroinflammation process.

### Astrogliosis, Neuroinflammation, and Consequences on Blood Perfusion and BBB

Astrocyte endfeet wrap the blood vessels and they play a key role in BBB properties in collaboration with pericytes (Zhang et al., 2012). Astrocytes become “reactive” and proliferate to form a glial scar in various severe brain injury models (Burda and Sofroniew, 2014). Depending on the timeline of the pathological process after injury, astrogliosis can be both beneficial and detrimental to the brain tissue adjacent to the lesion (Sofroniew, 2009). Astrocytes have various physiological functions such as providing energy substrates for neurons, regulating ion and neurotransmitter homeostasis, participating in synapse development and transmission, and regulation of CBF. Then, transformation of astrocyte in “reactive astrocyte” can have a direct and weighty impact on the brain functions on the post-injury outcomes (Sofroniew and Vinters, 2010). Astrocytes are among the first responder brain cells to TBI, and the mechanical forces of the primary injury trigger reactive astrocyte or astrogliosis (Burda et al., 2016). In fact, stretch injury or shear stress involves a rapid calcium influx in the astrocytes (Rzigalinski et al., 1998; Maneshi et al., 2015), by potential activation of astrocytic mechano-receptive channels (Burda et al., 2016). In parallel, activation of the astrocyte potentially contributes to the release of vasoactive substances (Howarth, 2014). In fact, release of isoprostanes (Hoffman et al., 1997, 2000) and endothelin 1 (Ostrow et al., 2000), powerful vasoconstrictors, have been described to increase after TBI in preclinical models (Petrov and Rafols, 2001; Armstead and Kreipke, 2011; Armstead and Raghupathi, 2011). Then, any changes in the calcium concentration within astrocytes could have a direct or indirect consequence on the regulation of CBF and perfusion post-injury. Then, calcium changes in astrocytes could contribute to the NVU dysfunction post-TBI with the increase of vasoconstrictor molecules.

Astrocytes play an important role in the integrity of the BBB (Abbott et al., 2006). Cytokines and inflammatory mediators are released by the astrocytes after injury with consequences on BBB properties (Chodobski et al., 2011; Sofroniew, 2014). For example, chemokines (Chodobski et al., 2011) and three isoforms of transforming growth factor–β (TGF- β) (Constam et al., 1992), potentially released by the astrocytes, have been implicated the BBB alterations after TBI (Shen et al., 2011). Very interestingly, increased TGF-β expression has been observed in patients with severe TBI where it parallels BBB dysfunction (Morganti-Kossmann et al., 1999). As already discussed, MMPs disturb the structure of the BBB after TBI and they are extensively produced by reactive astrocytes (Chen and Swanson, 2003). In the vicinity of the perivascular astrocyte endfoot, the water channel aquaporin 4 expression is altered after TBI, which is related to the formation of cerebral edema (Badaut et al., 2014; Gatto et al., 2015; Zhang et al., 2015). Increased aquaporin expression associated to edema brain formation event is also described in earlier stages of several neurodegenerative diseases (Sun et al., 2016; Yang et al., 2016; Gatto et al., 2018). There is a correlation between the level of aquaporin 4 expression and disruption of BBB (Fukuda and Badaut, 2012). The astrocyte endfeet play a critical role in the BBB integrity, and very interestingly, the physical interaction between astrocyte endfeet and the brain vasculature can also be altered after TBI (Villapol et al., 2014), with a direct contribution to the vascular dysfunction after injury.

Altogether, astrocyte phenotypic transformation can feed forward the brain-vascular pathology observed after TBI in relation to neuroinflammation and the complement pathway. In addition, the neurodegeneration proposed after TBI could be a result of astrocyte activation and chronic inflammation (Faden and Loane, 2015). Interestingly, astrocyte plays a potential role in immune response in the brain with expression of the specific receptors to complement proteins (see *part 3*). Therefore, it may be important to consider the contribution of reactive astrocytes into the long-term consequences of vascular dysfunction and a target of the activation of the complement pathway. This pathway could represent a new avenue for drug development targeting the NVU.

## General Description of the Complement Pathway

The complement system is part of an innate and adaptive immune response after infection acting as a primary defense and mechanism of clearance in order to preserve the organ against pathogens. It includes a wide collection of blood and cell surface proteins (C3, C5, CFB, CFD, CFH, C1q, etc.), whose activation results in a self-amplifying cascade of proteolytic reactions (Walport, 2001). Most of the plasmatic complement proteins (90%) are produced by the hepatocytes in the liver (C3, C5, CFB, CFH), released in the bloodstream to circulate as inactive forms. However, a local expression of complement proteins is also observed in cells (microglia/macrophages/astrocytes and neuronal cells) in the brain and the eye, mainly for C3 and its cleavage regulators (CFH and CFB).

**Figure 1:**
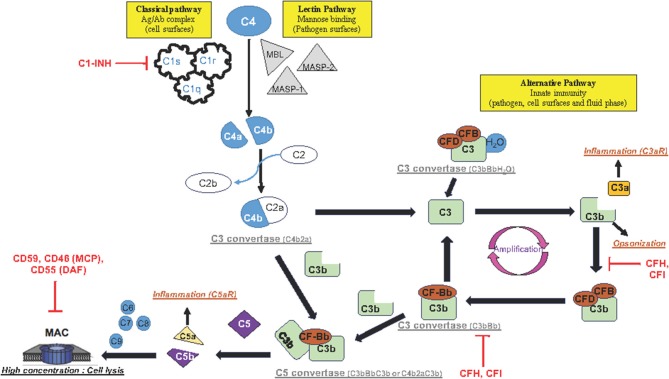
Schematic representation of complement activation pathways: The complement system is composed of three pathways (classical, lectin, and alternative pathway) that involve a collection of blood and cell surface proteins to eliminate pathogens from organism or damaged cells. This system consists of a series of proteins that interact with one another in a highly regulated manner. The classical pathway helps antibodies and phagocytic cells to clear pathogens or damaged cells. The lectin pathway is activated when MBL binds to mannose residues on the pathogen surface. This pathway is very similar to the classical pathway. The alternative pathway is an important part of innate immune system in which proteins cleave one another to form enzymatic complex able to initiate amplification of further cleavages and finally promotes inflammation by production of anaphylactic fragments. The final product of each activated complement pathway is the formation of the MAC in the target cell membrane. Once activated, the complement system has several major functions: lysis of pathogens or damaged cells, activation of inflammation, opsonization, and immune clearance.

The complement system is a key molecular pathway in various cellular and molecular activities, ranging from cell lysis to increase in the local inflammatory response. The complement activation is composed of three main pathways: classical, lectin, and alternate. They converge to the common effector pathway by forming the membrane attack complex (MAC) as illustrated in Figure 1.

### The Classical Pathway

The classical pathway activation is antibody-dependent. It happens when the complement protein C1 complex, formed by C1q, C1r, and C1s proteins, interacts with antigen–IgM or aggregated antigen–IgG complex, or is antibody-independent. It also happens when poly-anions (e.g., heparin, protamine, DNA and RNA from apoptotic cells), gram-negative bacteria, or bound C-reactive protein reacts directly with the C1 complex. Once the C1 complex is activated, then C1s cleave C4 and C2 into C4a/C4b and C2a/C2b, respectively, and forms the C3 convertase of the classical pathway (C4b2a). C4b2a cleaves C3 into C3a and C3b fragments. The classical pathway is regulated by C1 inhibitor (C1-INH).

### The Lectin Pathway

The lectin pathway is activated when mannose-binding lectin (MBL), a serum protein, binds to mannose or fructose groups on bacterial cell walls, yeast walls, or viruses, independently of the antibody complex. The lectin pathway shares similarities with the classical pathway.

### The Alternative Pathway

The alternative pathway is activated after cleavage of a small amount of complement compound C3 caused by microbial cell surfaces or immunoglobulin. This pathway is functionally present in physiological situation, with a spontaneous (hydrolysis) cleavage of C3 to C3a/C3b. The C3b product is rapidly inactivated by its binding to proteins complement factor H (CFH) and associated with complement factor I (CFI) as a co-factor, and then its opsonization functions (tagging pathogens, immune complexes apoptotic cells for phagocytosis) are aborted. After injury, C3b product binds to complement factor B (CFB) and its co-factor complement factor D (CFD) to form a molecular complex “C3bBb,” called C3 convertase complex, which acts on additional C3 to form more C3b and C3a products (Figure 1). This function of C3b contributes to a positive feedback loop and amplifies a first C3 cleavage that is induced by either the classical pathway or both classical and alternative pathways, in order to produce high-level C3b and C3a proteins. It is important to highlight that each complement pathway (classical, lectin, and alternative pathways) activation ends up into the cleavage of C3.

### Complement Cleavage Products Functions

Once activated, the complement pathway produces a high concentration of anaphylactic C3a/C5a and opsin C3b proteins (Figure 1). The receptors of these complement proteins (C3aR, C5aR, and CD11b, respectively) are expressed on cell membranes in various cerebral cell types such as endothelial cells, microglia/macrophage cells, and astrocytes. The wide distribution of the complement receptors underlines the variety of cellular functions to this system. Importantly, the complement system makes the link between the innate and acquired immunity by (a) enhancing antibody response and immunologic memory, (b) lysing the pathogens, and (c) clearing immune complexes and apoptotic cells.

The anaphylactic C3a molecule has a remote result because it diffuses away to play the role of an inflammation and chemotactic factor. In contrast, the produced C3b binds to the C3 convertase complex (C3bBb) to form a C5 convertase complex (C3bBbC3b). The C5 convertase complex is located in the cell membrane or in the bloodstream and cleaves complement 5 (C5) into C5b and C5a fragments. Similarly to C3a, the C5a product is described in the bloodstream to act remotely as an anaphylactic protein inducing a general inflammatory reaction. In contrast, C5b initiates the MAC in the cell membrane (Figure 1). Sequentially, C5b interacts successively with C6, C7, and C8 complement proteins, and finally with a number of C9 complement proteins to form MAC. Importantly, MAC is organized in a circle with, in its center, a pore that produces a hole in the cell membrane. High concentration of MAC induces lysis of the foreign cell (such as microbes or bacteria) or apoptosis (of host cell).

Very interestingly, MAC has been found to contribute to secondary damages in various preclinical models of human brain diseases (Leinhase et al., 2006; Stahel et al., 2009; Fluiter et al., 2014; Ruseva et al., 2015). However, depending on the level of expression of MAC, opposite physiological responses can be observed. In fact, a low level of the MAC (C5b9) complex on the cell surface promotes cell-cycle progression instead of cell lysis or apoptosis (Fosbrink et al., 2005). The role of MAC in cell-cycle progression is due to the transactivation of receptor tyrosine kinases, a regulator of cytosolic free Ca^2+^ concentration (Cybulsky et al., 1999), and activation of protein kinase C and cytosolic phospholipase A_2_-α (Cybulsky et al., 1995; Panesar et al., 1997).

It is important to have a fine-tuned regulation of MAC formation with a full panel of specific inhibitor proteins. Many of the complement regulator proteins are found in the blood plasma and on the cell membranes, and among them, CFH acts on the activity of the upstream C3 convertase complex (C3bBb) in the blood and on the cell surface. CFH removes “Bb fragment” from the C3 convertase enzymatic complex and inactivates C3 cleavage by competition with CFB for binding C3b; it stops the main amplification loop of the alternative complement pathways (Figure 1). Downstream, CD55 (or DAF: decay accelerating factor), CD46 (or MCP: membrane cofactor protein), and CD59 (known as protectin) have also been described as inhibitors of MAC formation in the cell membrane for both classical and alternative complement pathways (Figure 1).

## Involvement of Complement Pathway in TBI Pathophysiology

The secondary injury events post-TBI include inflammatory mechanisms with early activation of the innate immune system, and then the chronic inflammation post-injury possibly contributes to the progressive neurodegenerative process (Francis et al., 2003; Schmidt et al., 2005; Stahel and Barnum, 2006).

The complement pathway represents a key element in the inflammatory response, and it contributes to neuronal cell death, edema, and infiltration of inflammatory cells after injury using rodent models of moderate and severe TBI (Kaczorowski et al., 1995; Ruseva et al., 2015; Rich et al., 2016). Involvement of the complement pathway observed in rodent TBI models has been confirmed with up-regulation of CFB (Kossmann et al., 1997), C3, C1q, and C4 (Stahel et al., 1998), and increase of soluble terminal complement complex C5b9 has been detected in ventricular cerebrospinal fluid (CSF) of severe TBI patients for up to 10 days after trauma, possibly due to the alteration of the BBB integrity (Stahel et al., 2001). CSF increase of complement proteins is paralleled by higher concentration of complement proteins in blood plasma of TBI patients (Manek et al., 2018). Expression of complement protein C3 and C9 has been described up to 6 months post-injury (Bao et al., 2018) in patients, suggesting a continuous complement pathway activation in the chronic TBI. Very interestingly, similar observations have been found in various preclinical rodent models, with higher levels of C9 and CFB in serum of rat during the first days after TBI (Thelin et al., 2018). Deposits of C3 have also been described in the peri-lesion tissue, while C9 protein has been reported in damaged neurons after cortical contusion in the adult rat (Bellander et al., 1996). Thus, a similar change in activation of the complement pathway has been described in clinical and in preclinical rodent models.

As presented above, C3 protein is common to the three complement pathways. In this line with the complement pathway inhibition in TBI, C3^−/−^ mice exhibit less brain edema and microglia activation/infiltration compared to WT after TBI, suggesting a role of the complement pathways in the secondary injury cascade (Sewell et al., 2004; Yang et al., 2006) and inhibition of C3 could be a therapeutic strategy. Interestingly, severe TBI swine resuscitation with valproic acid (a histone deacetylase inhibitor) induces a down-regulation of the complement system activation, resulting in a significant decrease of lesion size after resuscitation (Dekker et al., 2014a,b; Bambakidis et al., 2016). Similarly, inhibition of MAC has been proposed to be protective. In fact, over-exuberant MAC formation contributes to neuronal cell death after severe TBI injury in mice lacking CD59, an inhibitor of MAC formation on the membrane cells (Figure 1) (Stahel et al., 2009). The inhibition of MAC formation using C6 antisense oligonucleotide or an inhibitor of C5 convertase complex reduces not only MAC deposition but also accumulation of microglia/macrophages, neuronal death, and axonal loss and enhances neurologic performances as compared to the placebo-treated TBI group (Fluiter et al., 2014).

The current paradigm is that MAC formation is predominantly implicated in development of post-TBI process neuropathology and neuroinflammation, but it is not excluded that C3 cleavage inhibition could prevent inflammation and/or cell death. Then, C3 and MAC would be good therapeutic targets to reduce TBI damage. Studies show that inhibition of C3 cleavage reduces post-injury of microglial and astrocyte activations, C3 deposits, and the extent of neuron cell death, and then triggers improvements in cognitive and functional recovery (Rich et al., 2016; Alawieh et al., 2018). It demonstrates a critical role of the complement system and mainly of the alternative pathway in the TBI neuropathology process.

Targeted deletion of *cfb* gene expression, the activator of alternate complement pathway by activation of C3 convertase, in transgenic mice clearly reveals a significant decrease of neuronal cell death post-TBI (Leinhase et al., 2006). In support to these results in transgenic mice, inhibition of the CFB by injection of anti-CFB monoclonal antibody attenuates both inflammation and cell death in TBI animals (Leinhase et al., 2007), confirming a key role of the alternative complement system in TBI.

Both the classical and lectin pathways have also been assessed in preclinical TBI models and human studies: (1) C1-inhibitor injection after TBI mitigates motor deficits, cognitive dysfunction, and contusion volumes (Longhi et al., 2009), arguing for a role of the classical complement pathway in TBI secondary injuries. (2) Activation of the lectin complement pathway has been shown to be involved in secondary ischemic/inflammatory injury after severe TBI along with an increase of MBL protein in plasma of patients at 48 h post-injury (Osthoff et al., 2017). However, there is no correlation between lectin complement pathway activation and mortality/consciousness after severe TBI (Osthoff et al., 2017). In human and mice, MBL is expressed in the injured cortex from 30 min and persists up to 1 week post-TBI (Longhi et al., 2014). The inactivation of the lectin complement pathway induces a decrease of cortical cell death (Longhi et al., 2014), arguing that this deletion has a protective effect in TBI damages. Until now, no specific pharmacological compound has been found to limit TBI damages, but targeting complement pathway would be a novel therapeutic way.

## Chronic TBI and Neurodegenerative Landmarks

### Neurodegenerative Landmarks After TBI

Brain vasculature alterations and inflammatory processes implicating complement pathways are very likely hand in hand in the early secondary events post-injury. Several works clearly indicate that TBI is a chronic brain disorder with development of neurodegenerative disease landmarks such as accumulation of the amyloid-β (Aβ) (Johnson et al., 2010; Pop and Badaut, 2011; Pop et al., 2013). We and other groups have shown changes in the brain vasculature and their involvement in functional outcome after TBI, with BBB dysfunctions several months and years after the injury (Pop et al., 2013; Hay et al., 2015). We previously described increase of claudin-5 expression in large blood vessels, decrease of the efflux pump P-glycoprotein (P-gp), and increase of the perivascular matrix proteins perlecan and fibronectin up to 6 months post-injury in a juvenile TBI model (Pop et al., 2013; Jullienne et al., 2014). The matrix changes observed up to 6 months after juvenile TBI (Jullienne et al., 2014) have also been described in AD patients (Lepelletier et al., 2017). Changes in the matrix properties possibly participate in neurodegenerative processes by leading to the accumulation of Aβ, decreasing its clearance and its perivascular drainage (Pop et al., 2013; Jullienne et al., 2014). P-gp has been suggested to be a key player in Aβ clearance from the brain parenchyma. P-gp knock-out models have increased Aβ deposition after injection of Aβ in the brain compared to the wild type (Cirrito et al., 2005). Moreover, P-gp expression is decreased on endothelial cells in aged human and AD brains as well as in aged rodents (Silverberg et al., 2010). In addition to the vascular dysfunction, the presence of Aβ deposition could contribute to amplify the pathophysiology with activation of the complement pathways around the Aβ deposition, and the vicious cycle of chronic inflammation would accelerate the brain aging.

### Complement Pathway Activation in Neurodegenerative Disease

A number of chronic neurodegenerative diseases of the central nervous system (CNS) are characterized by prominent neuroinflammation marked by activated astrogliosis and microgliosis. In AD and MS affections of CNS, similar to what is seen in TBI, there is strong evidence of complement involvement in the pathogenesis that arises from a confluence of histochemical, genetic, and model data. The involvement of the complement system in the AD process comes from the identification of complement receptor 1, the C3b receptor expressed on macrophage/erythrocyte or monocyte cells, as a genetic risk to develop this neurodegenerative disease (Lambert et al., 2009; Crehan et al., 2012). In fact, erythrocyte cells capture complement-opsonized Aβ through CR1 receptor expressed on their membrane and facilitate clearance mechanism, which is relevant in AD pathophysiology (Brubaker et al., 2017). On the other hand, Aβ deposition promotes complement pathway activation by inhibition of CFI enzymatic activity, a cofactor of CFH (Lashkari et al., 2018). Thus, accumulation of Aβ contributes to increase C3 complement protein and ends up giving rise to the anaphylactic C3a/C5a proteins and MAC formation (Bradt et al., 1998; Lian et al., 2015). In fact, accumulation of these complement proteins generates damaged neurons, increases numbers of activated glial cells (Bradt et al., 1998), and, through neuronal C3a/C3aR signaling, disrupts dendritic morphology and network function (Lian et al., 2015). The Aβ-induced neuroinflammation response is also exaggerated by C5a/C5aR signaling (An et al., 2018), demonstrating another link between Aβ and complement activation. Similarly, the role of the complement pathway has been investigated in the context of the neuroinflammatory autoimmune neurodegenerative disease MS. Comparatively, these studies would help to understand the potential role of complement pathways in the long-term outcomes after TBI. With the aim to better understand the cause of MS, a demyelinating and neurodegenerative disease of the CNS, several investigations defined and quantified complement compounds in the blood circulation and CSF. In relation to the inflammatory activity of MS, to define potential biomarkers of the various MS disease forms, levels of C3, C4, and C9 were mostly investigated, providing convincing evidence of the role of complement compounds in MS disease development. The data obtained from various cohorts of patients, however, generated controversies. Indeed, early studies in patients with progressive MS revealed elevated levels of C4 in MS-serum and reduced C4 levels in MS-CSF (Jans et al., 1984). These findings were, however, not supported by others (Jongen et al., 2000). A more recent study defined elevated plasma C4a levels and elevated C3 levels in CSF of patients with active relapsing-remitting MS (RRMS) potentially providing furthermore follow-up markers for therapeutic efficacy (Tatomir et al., 2017). The levels of C9 compound and terminal complement complex (MAC) in CSF and/or blood also give heterogeneous quantitative data found to be reduced, unchanged, or increased (Ingram et al., 2009). Differences in the presence of the complement compounds in the CSF and blood circulation might be related to the modulation of the BBB by the inflammatory disease activity at the various disease MS forms. Histophathological post-mortem investigations of MS brain tissues revealed complement deposition and activation. Activated microglia is commonly observed in contact with axons suffering from demyelination. In brain tissue from patients with progressive MS, a combined immunohistopathology and *in situ* hybridization study showed the local expression of compounds of the complement activation pathways. Both transcripts for C1qA, activation fragment and opsonin C3b, and immunostained proteins C1q of the classical complement pathway and the activation CFB-fragment Bb of the alternative complement pathway were observed in neurons and/or glia within and in the vicinity of cortical and deep gray matter lesions. Furthermore, activated microglia expressing complement *anaphylatoxin receptor* were observed in these lesions in post-mortem brain tissue (Watkins et al., 2016). The deposits of activated C3 fragment on microglia in such chronic cortical lesions, but not in acute disease, indicate that C3 could function as a mediator for the removal of damaged nervous cells in the MS animal model of the relapsing experimental autoimmune encephalomyelitis (EAE) (Michailidou et al., 2017). The formation of MAC as a final complement product has been reported in acute and chronic white matter lesions of MS and would have a direct impact on nervous tissue destruction (Compston et al., 1989; Storch et al., 1998). Many experimental animal studies of MS support the complement-mediated role of nervous tissue degeneration. Further evidence for MAC in the inflammatory disease progression is documented in the EAE mouse model by the systemic curative administration of MAC inhibitors that prevents relapses after a first clinical attack (Michailidou et al., 2018). In EAE gray matter neurodegeneration, a significant synapse loss in the CA1 statum radiatum of the hippocampus reflecting cognitive impairment is caused by increased complement production and deposition of Cq1 and C3d that could make synapses vulnerable to phagocytosis by microglia. In particular, C3 mediates microglial activation and EAE motor impairments (Hammond et al., 2019). Altogether, there is convincing evidence that the complement activation is involved in MS disease development and the cerebral complement over-activation in inflammatory CNS lesions may be essential for the irreversible progression of MS. The knowledge gathered on the role of the complement pathway activation in MS patients and animal model of MS can be useful for understanding the chronic brain disorders post-TBI. The presence of the complement in serum can serve as a biomarker in TBI patients, and the potential inhibition of the complement can be a therapeutic strategy for the long-term consequences of TBI.

## Concluding Remarks

Complement pathway activation is present early after TBI and can contribute to the development of the secondary injuries by inducing neuronal cell loss and synapse pruning (Table 1). Very likely, the complement activation is still present in the long term after TBI (Table 1). The knowledge on complement activation in AD and MS strongly suggests that the activation of the complement pathways in the long term after TBI could be an interesting mechanism in order to develop new therapeutic approaches for chronic TBI. It is possible that the pruning of synapses by complement-mediated microglia activation causes long-term neurological handicaps including cognitive impairment in various models of neurodegenerative diseases such as Alzheimer's disease, viral encephalitis, TBI, and MS and potentially contributes to schizophrenia (Stevens et al., 2007; Paolicelli et al., 2011; Ingram et al., 2014; Hong et al., 2016; Sekar et al., 2016; Vasek et al., 2016; Alawieh et al., 2018; Hammond et al., 2019). Therefore, inhibition of the long-term complement activation could improve TBI outcomes.

**Table 1:** Summary of complement effects on the TBI process in animals models and human studies.

**Human**	**Complement analysis**	**Complement levels**	**References**
Severe TBI	C3/CFB	↑CSF	Kossmann et al., 1997
Severe TBI	C3/C1q/C4	↑CSF	Stahel et al., 1998
Severe TBI	C5b9	↑CSF	Stahel et al., 2001
Severe TBI	C3/C9	↑Plasma	Bao et al., 2018
Severe TBI	MBL	↑Plasma	Osthoff et al., 2017
**Animal models**	**Complement target**	**Complement biologic effects**	**References**
Cortical impact (severe TBI)	C9/CFB	↑Serum	Thelin et al., 2018
Cortical contusion (severe TBI)	C3	↑Deposits (peri-lesion tissue)	Bellander et al., 1996
Cortical contusion (severe TBI)	C9	↑Deposits (damaged neurons)	Bellander et al., 1996
Intra-cerebral hemorrhage	*C3*^−/−^ mice	↓Brain edema ↓Microglia activation/infiltration	Sewell et al., 2004; Yang et al., 2006
Severe TBI swine resuscitation	CD59/C1q	↓Complement system activation associated to a decrease of lesion size	Dekker et al., 2014a,b; Bambakidis et al., 2016
Cortical impact (severe TBI)	CD59^−/−^ mice	↑Neuronal cell death	Stahel et al., 2009
Severe closed head injury mice	C6 antisense oligonucleotide Or C5 convertase inhibition	↓C5b9 deposits/axon loss ↓Microglia/macrophage accumulation ↓Neuronal cells death ↑Neurologic perfomances	Fluiter et al., 2014
Cortical impact (severe TBI)	C3 cleavage inhibition	↓Microglial/astrocyte activations ↓C3 deposits/neuron cells death ↑Cognitive/functional recovery	Rich et al., 2016; Alawieh et al., 2018
Focal trauma (severe TBI)	*cfb*^−/−^ mice	↓Neuronal cell death	Leinhase et al., 2006
Focal trauma (severe TBI)	Anti-CFB antibody	↓Inflammation/cell death	Leinhase et al., 2007
Cortical impact (severe TBI)	C1q inhibitor	↓Cognitive dysfunction/contusion volumes	Longhi et al., 2009
Cortical impact (severe TBI)	MBL	↑MBL deposits (injured cortex)	Longhi et al., 2014
Cortical impact (severe TBI)	*mbl*^−/−^ mice	↑Cortical cell death	Longhi et al., 2014

*CSF, CerebroSpinal Fluid; MBL, Mannose Binding Lectin protein*.

## Author Contributions

VD and JB contributed to the design and concept of the review manuscript. VD, KP, and JB wrote and revised the manuscript.

### Conflict of Interest

The authors declare that the research was conducted in the absence of any commercial or financial relationships that could be construed as a potential conflict of interest.
